# Recent advances in esophageal squamous cell precancerous conditions: A review

**DOI:** 10.1097/MD.0000000000032192

**Published:** 2022-12-16

**Authors:** Tianjiao Wen, Wei Wang, Xinran Chen

**Affiliations:** a Pharmacy Department, the Fourth Hospital of Hebei Medical University, Shijiazhuang, Hebei, PR China; b Department of clinical laboratory, Hebei General Hospital, Shijiazhuang, Hebei, PR China.

**Keywords:** biomarkers, endoscopy, Esophageal squamous cell carcinoma, precancerous condition

## Abstract

Esophageal squamous cell carcinoma (ESCC) is a common cancer in many developing countries in Asia and Africa, with a 5-year survival rate of approximately 20%. Most cases are diagnosed at an advanced age when there is no effective treatment strategy. Esophageal precancerous conditions have a much better prognosis, with a 5-year survival rate of over 90% by endoscopic diagnosis and treatment. Nevertheless, limitations, contraindications, and lymph node metastasis incompetency of endoscopy. Thus, the diagnosis and treatment of esophageal precancerous lesions remain a significant challenge. Biomarker investigations provide opportunities for target detection and therapy. Additionally, drug development is ongoing. Changes in lifestyle habits, such as diet balance, smoking and alcohol cessation, are beneficial for the prognosis of esophageal precancerous lesions. Collectively, multiple and sequential diagnoses and treatments are essential for curing esophageal precancerous lesions and reducing the incidence and mortality of ESCC.

## 1. Introduction

Esophageal cancer is the eighth most common cancer and sixth leading cause of mortality cancer worldwide.^[1]^ Esophageal cancer has 2 histological types: squamous cell cancer and adenocarcinoma cancer.^[2]^ Although the incidence of adenocarcinoma cancer has increased rapidly in Western countries during the last 30 years,^[3]^ esophageal squamous cell cancer (ESCC) remains the biggest threat in Asia, Southern and Eastern Africa, and Northern France.^[4]^ In China, 90% of esophageal cancer cases are ESCC.^[5]^

Several risk factors are associated with ESCC. It has been proven that alcohol and tobacco consumption are strong risk factors for ESCC.^[6]^ Alcohol is an irritant to the esophageal epithelium, facilitating its susceptibility to other carcinogens. Smokers have a 5 to 10-fold higher risk than nonsmokers, with a possible mechanism by which tobacco induces gene damage, such as mutations, to epithelial cells.^[7]^ It is generally believed that smoking dose and exposure duration are positively correlated with P53 mutation in ESCC patients. Additionally, poor nutrition, such as fresh vegetables and fruit deficiency, excessive intake of preserved or fungal-contaminated food, is associated with a higher risk of ESCC.^[8]^ Genetic susceptibility is a risk factor for ESCC. Garavello reported that the odds ratio of family history of esophageal cancer in Italy and Switzerland was 3.2 in a first-degree relative, adjusted for smoking and alcohol consumption.^[9]^ Likewise, the incidence of ESCC is higher in males than females in most countries and higher in black men than in white men in the United States.^[10]^ ESCC is also correlated with human papillomavirus infection, poor oral hygiene, and old age.^[10]^

Surgery remains the mainstay treatment for ESCC.^[11]^ Although the surgical mortality rate is less than 5%, the long-term effect is still unsatisfactory.^[12]^ Some patients with lymph node metastasis should be treated by combining radiotherapy and chemotherapy.^[13]^ However, the 5-year survival rate of ESCC is only around 20%^[14]^ because most cases are diagnosed at an advanced stage with no effective treatment strategies.^[15]^ Esophageal precancerous conditions, the intermediate state of ESCC transforming from normal esophagus, however, have a significantly better prognosis, with a 5-year survival rate above 90% with proper treatment in time.^[16]^ Therefore, timely diagnosis and treatment of esophageal precancerous lesions may become a life-saving strategy to reduce the incidence and mortality of ESCC. In this study, the advancement of esophageal squamous cell precancerous conditions was reviewed.

### 1.1. Esophageal precancerous condition

A precancerous condition is a tissue abnormality in which atypical cells accumulate in the epithelium.^[17]^ According to the American National Cancer Institute conference in 2004, precancerous lesions are continuous and long-term changes that have detectable molecules and phenotypic qualities of the corresponding cancer induced by a series of internal and external risk factors.^[18]^ Studies have indicated that ESCC develops from a progressive sequence from mild, moderate, and severe dysplasia to carcinoma in situ (CIS) and finally to invasive carcinoma.^[19]^ In 1989, Takiyama evaluated 40 esophageal specimens using hematoxylin-eosin (HE) staining. In this study, precancerous lesions were classified as mild, moderate, or severe dysplasia.^[20]^ Mild dysplasia showed that the mucosal epithelium became thick, the lower one-third of the cells lost polarity, and the cell nucleus became enlarged and hyperchromatic. Moreover, moderate and severe dysplasia indicated that the epithelial layer became thicker, and less than one-half to three-fourths of cells lost polarity, with the cell nucleus enlarging more seriously. Additionally, CIS was finally formed when whole epithelial cells lost polarity and were immature. In the 2010 WHO classification, the term “intraepithelial neoplasia” was introduced to describe dysplasia and CIS. According to this classification, low-grade intraepithelial neoplasia (LGIN) includes mild or moderate dysplasia. In contrast, high-grade intraepithelial neoplasia (HGIN) substituted for severe dysplasia and CIS, 2 lesions with the same clinical implications.^[21]^

### 1.2. Diagnosis of esophageal precancerous lesions

In a sense, it is challenging but meaningful to diagnose esophageal precancerous conditions since they are almost asymptomatic. Currently, squamous dysplasia and early cancer are characterized by distinctive endoscopic changes, mucosal friability, focal red areas, erosion, plaques, and nodules. Dye agent staining-assisted endoscopy can reveal the degree, range, and number of lesions. Therefore, the detection was enhanced using Lugol’s iodine and toluidine blue.^[22]^ Additionally, endoscopy with high-definition television narrow-band imaging improves the definition,^[23]^ and confocal laser endomicroscopy can provide subcellular resolution images of esophageal lesions in vivo.^[24]^ Normalized autofluorescence imaging can also precisely distinguish tumorous mucosa from regular areas precisely.^[25]^

At present, there are no uniform guidelines for endoscopic screening for esophageal cancer, and with the continuous application of endoscopic screening, new issues need the necessary attention. First, there is no unified quality control standard for the detection rate, and the detection rate of esophageal cancer and precancerous lesions is uneven in each detection unit. The detection rate is closely related to the prevalence of monitoring points, screening compliance, histopathological diagnosis level, mastery of endoscopic screening technology and other factors. The lack of objective quality control standards will restrict the quality assessment of early diagnosis and treatment items of esophageal cancer screening. With the development of modern molecular biology techniques, various molecular markers offer the possibility to build efficient risk prediction models. If the effective protein markers can be screened out, especially the highly sensitive and specific biomarkers, they can be used to predict the disease and progression of precancerous lesions. Therefore, we further summarize the possible biomarkers of esophageal precancerous lesions as follows.

### 1.3. Biomarkers of esophageal precancerous lesions

Biomarkers are biological substances secreted or released from cancer cells or new genesis from the host, which can be used for tumor diagnosis, disease course and prognosis analysis, treatment guidance, and recurrence or metastasis detection. To further investigate the possible pathological mechanism of ESCC and explore additional biomarkers of esophageal precancerous conditions, the abnormal expression of these molecules in esophageal precancerous tissues or sera of pertinent patients was reviewed.

First, the factors that affect cell proliferation and cell cycle play an essential role in the malignant changes of the esophagus.^[26]^ Cyclin D1, a type of cyclin that promotes the cell cycle and accelerates cell proliferation, is overexpressed in many types of tumor tissues.^[27]^ Shamma et al investigated cyclineD1, cyclin-dependent kinase 4 inhibitor p16INK4, and cell cycle regulatory protein p27KIP1 expression in 36 squamous dysplasia and 34 early squamous cell carcinomas using immunohistochemical assays.^[28]^ These results indicated that cyclin D1 overexpression started early in dysplasia and could be a valuable marker for its malignant potential, while the reduction in p16INK4 and p27KIP1 occurred during the transformation from dysplasia to cancer. In addition, the proliferation of cell nuclear antigen, a cell cycle-dependent protein that promotes cell proliferation,^[29]^ increased from moderate dysplasia to invasive mucosal carcinoma and was significantly correlated with the expression of cyclin D1, p16INK4, and p27KIP1.^[28]^

The Ki-67 protein is a cellular marker for proliferation and is expressed positively in many pathological tumor grades. It is present during all active phases of the cell cycle, but is absent in resting cells.^[30]^ ProExC is an immunohistochemical surrogate marker that targets the cell cycle proteins minichromosome maintenance protein-2 and topoisomerase II-alpha, resulting in increased levels of aberrant S-phase induction.^[31]^ Strong Ki-67 and ProExc staining was observed in esophageal squamous dysplasia. Ki-67 may be a good biomarker to distinguish HGIN from LGIN because it is expressed explicitly on HGIN but not on LGIN, and strong ProExC staining was also observed in 80% of HGIN.^[32]^

As we know, the abnormal expression of P53 and P63 is closely related to tumorigenicity and development.^[31]^ Yasuda et al reported abnormal accumulation of the p53 protein at precancerous stages, such as basal cell hyperplasia or dysplasia of the esophagus, suggesting that p53 mutations are essential even in the early stage of esophageal squamous cell carcinogenesis.^[33,34]^ Abnormal P63 expression is upregulated in squamous neoplastic conditions and may contribute to squamous carcinogenesis.^[35]^

Additionally, P16^[36]^ is a tumor suppressor gene product, while C-myc^[37]^ is a cancer gene protein. CyclinB1^[38]^ is a periodic protein that plays a regulatory role in mitosis. P62^[39]^ and insulin-like growth factor II mRNA-binding protein 1 (IMP1)^[40]^ are 2 RNA-binding proteins expressed in cancer. Zhou et al studied tumor-associated antigens P53, IMP1, P16, cyclin B1, P62, and C-myc for screening high-risk subjects and early detection of ESCC.^[41]^ The autoantibody assay successively accumulated 6 antigens. A stepwise increase in the detection frequency of autoantibodies was found:6% in normal, 18% in basal cell hyperplasia, 38% in dysplasia, and 64% in ESCC, and the risks to basal cell hyperplasia, dysplasia, and ESCC steadily increased about 3-, 9-, and 27-folds.

The abnormal expression of some transcription factors correlates with esophageal malignancy. For example, early growth response gene-1 (*Egr-1*) is a transcription factor that transmits information across the nuclear membrane. It can be rapidly induced and then initiates a series of downstream target genes in all cell types.^[42]^ Wu et al studied *Egr-1* expression in human ESCC and precancerous lesions using in situ hybridization, immunohistochemistry, and TUNEL tests. The data showed that Egr-1 mRNA and protein expression was high in precancerous lesions of the esophagus.^[43]^

*PAX* genes encode a family of transcription factors that play crucial roles in oncologic development^[44]^ The paired box 9 (PAX9) protein serves as a transcription factor responsible for crosstalk between epithelial and mesenchymal tissues.^[45]^ Gerber et al investigated *PAX9* expression in esophageal dysplasia and cancer tissues.^[46]^ The results indicated that the percentage of PAX9-positive cells within the epithelium decreased with increasing malignancy of the epithelial lesion. Additionally, data was identified *PAX9* as a sensitive marker for the deregulated differentiation of esophageal keratinocytes and indicated a role for *PAX9* in the normal differentiation process of internal stratified squamous epithelia.

Xu et al investigated transforming growth factor β1 and hepatocyte growth factor (HGF) protein secretion by esophageal squamous epithelial cells and stromal fibroblasts in esophageal carcinogenesis using immunohistochemical assay.^[47]^ Results showed that the expression levels of alpha-smooth muscle actin, transforming growth factor β1, and HGF increased significantly in the order of normal, LGIN, HGIN, and ESCC. Additionally, linear correlations were observed between the expression of the 3 proteins and different lesions.

Notch1 is a transmembrane receptor that determines cell fate.^[48]^ Sakamoto et al discovered that Notch1 downregulation was observed in precancers, and that there was little difference between ESCC and HGIN. In culture experiments, reduction of Notch1 expression resulted in the downregulation of keratin 13 and keratin 15, and upregulation of keratin 17. Notch1 knockdown cells formed a dysplastic stratified epithelium mimicking precancerous lesions. These data indicate that the reduction of Notch1 expression directed the basal cells to cease terminal differentiation and to form an immature epithelium, thereby playing a major role in the histopathogenesis of epithelial dysplasia.^[49]^

Methylenetetrahydrofolate reductase 677T (MTHFR 677T) accelerates the conversion of 5, 10-methylene methylenetetrahydrofolate to 5-methyl methylenetetrahydrofolate. Consequently, this enzyme plays a key role in balancing the pool of methyl groups during DNA synthesis and methylation.^[50]^ Huang et al analyzed the association between the MTHFR C677T polymorphism and esophageal cancer risk using the polymerase chain reaction-restriction fragment length polymorphism method.^[51]^ They found that the MTHFR 677T genotype distribution in the moderate or severe esophageal precancerous lesion groups and the T allele in the moderate group were significantly different from those in the mild group. Therefore, MTHFR C677T may be a potential biomarker for identifying precancerous lesions and various stages of pathological precancerous conditions.

Recently, ubiquitination was shown to be a versatile post-translational modification that regulates different aspects of cellular physiology by ubiquitylating and deubiquitylating enzymes. Ubiquitin-specific peptidases (USPs), the largest group of deubiquitylating enzymes, play a fundamental role in the ubiquitin system by specifically deconjugating ubiquitin from ubiquitylated substrates. Ubiquitin-specific peptidase 9 (USP9X), a member of the USP family, is involved in multiple physiological pathways by targeting various substrates. Elevated USP9X expression at the translational level correlates with poor prognosis in multiple myeloma and ESCC.^[52]^ Jing *et al* reported that USP9X expression is correlated with tumor progression and poor prognosis in ESCC.^[53]^ The expression of USP9X was found to be significantly different between normal mucosa and ESCC and between LGIN and HGIN. However, no difference was observed between the high expression of USP9X in normal mucosa and LGIN or between HGIN and ESCC. Therefore, USP9X may be a promising marker for differentiating LGIN from HGIN.

Plant-associated human cancer antigen (PHCA) is a transcriptional regulator that activates the expression of multiple virulence genes in the plant pathogen *Ralstonia solanacearum*.^[54]^ Jun et al studied the relationship between the expression profiles of PHCA and esophageal carcinogenesis and its significance.^[55]^ There was a significant difference in expression between the normal mucosa and severely dysplastic tissues or CIS. A significant difference was also observed between mild and severe dysplasia and CIS.

The fragile histidine triad (*FHIT*) gene is one of the earliest and most frequently altered genes in most human cancers.^[56]^ Recent reports have reconfirmed *FHIT* as a tumor suppressor gene with roles in apoptosis and prevention of epithelial-mesenchymal transition, which *FHIT* leads to nucleotide imbalance, spontaneous replication stress, and DNA breaks.^[56]^ The loss of *FHIT* expression induced by heavy smoking and alcohol consumption occurred during esophageal precancerous lesions and early cancer stage.^[57]^

Telomerase is a reverse transcriptase enzyme that is a type of DNA (RNA)—dependent nucleic acid-protein complex.^[58]^ The activation of telomerase keeps the length of the telomere stable, which allows the cell to obtain the ability of unlimited proliferation and thus become immortal cells. Telomerase activity is associated with the development of most human malignancies.^[59]^ Overexpression of telomerase may be an early event in carcinogenesis in the esophagus, which affects the transformation of esophageal lesions from benign lesions to malignant tumors.^[60]^

Squamous cell carcinoma antigens (SCCAs) are intracellular serine protease inhibitors. Both SCCA1 and SCCA2 are normally detected in the cytoplasm of squamous epithelial cells and inhibit serine proteases of endogenous origin, such as cathepsin G and chymase, and extrinsic origin, such as cysteine proteases.^[61]^ Yang reported that SCCA2 mRNA expression in the peripheral blood is linked to different stages of esophageal pathological changes. This knowledge may help monitor the processes of change that occur in premalignant esophageal lesions among subjects who live in a high-incidence area.^[62]^

Altogether, CyclinD1, Egr-1, PAX9, alpha-smooth muscle actin, TGF-β1, HGF, Notch1, P53, P63, PHCA, and FHIT was significantly correlated with different stages of precancerous conditions. In contrast, high proliferation of cell nuclear antigen expression and MTHFR 677T abnormal distribution are mainly involved in moderate and severe dysplasia. In addition, P16INK and P27KIP1 expression was reduced during the transformation from esophageal dysplasia to ESCC. Furthermore, telomerase activation is a significant risk factor for normal esophageal malignancies. Notably, the SCCA-2 antigen levels increased step-by-step in esophageal precancerous and cancerous patients. Autoantibodies of P53, IMP1, P16, Cyclin B1, P62, and C-myc in the sera of patients with esophageal precancerous conditions or ESCC also increased markedly. More importantly, the expression of biomarkers at different stages of esophageal precancerous lesions is conducive to the diagnosis of pathological grade and provides a theoretical basis for combing and sequential therapy for multiple targets.

Here, it can be speculated that the application of biomarkers in diagnosing esophageal precancerous lesions will play an increasingly important role. In particular, the detection of markers in sera is a convenient, fast, and inexpensive diagnostic method. Multiple indices combining detection are necessary to improve accuracy and specificity. Fortunately, serum surface-enhanced laser desorption/ionization-time of flight mass spectrometry, a high-throughput technology for identifying cancer biomarkers using serum drops, meets these tests. One or more biomarkers can be simultaneously tested with the instrument. Zhai et al reported that types of precancerous lesions can be distinguished with an accuracy of 95.2% (dysplasia), 96.6% (basal cell hyperplasia), and 93.8% (healthy controls), with a biomarker of 25.1-kDa.^[63]^ The simultaneous detection of multiple markers can further improve the sensitivity and specificity of the assay.^[64]^

We hope to establish a highly sensitive and specific detection model based on the combination of endoscopy and tumor markers, so as to make the detection of precancerous lesions of the esophagus more accurate and efficient.

### 1.4. Treatment of esophageal precancerous lesions

The outcomes of precancerous conditions include discrepancy, differentiation into normal tissue, maintenance, or advancement to cancer tissue.^[18]^ Alternatively, precancerous lesions are more prone to developing cancer than adjacent normal tissues and other benign lesions. However, the progression from precancerous lesions to cancer is continuous and long-term, and some risk factors have a continuous influence. However, cancer is not the only outcome since some precancerous lesions may not progress to cancer in the long term. Some precancerous conditions may regress to normal tissues if the risk factors disappear. Therefore, the choice of treatment plan for esophageal precancerous lesions should be based on the situation.

Generally, LGIN is considered less likely to develop into cancer in the short term with a chance to differentiate into normal conditions, despite no medicine presently facilitating differentiation.^[65]^ Thus, LGIN are commonly observed regularly. HGIN, cancerous tissues that occupy more than half of the epithelium, but do not invade the muscular layer, are principally excised using endoscopy. Conventional white-light endoscopy has a miss rate of up to 25% for gastrointestinal pathology and, therefore, must exit the stage of history.^[66]^ Chromoendoscopy is a common endoscopy in esophageal precancerous lesionectomy.^[67]^

Additionally, chromoendoscopy combined with magnifying endoscopy can further increase resolution and improve the treatment effect. New esophageal endoscopy techniques include photodynamic therapy and endoscopic argon plasma coagulation treatment. 5-aminolevulinic acid is the main photosensitizer of photodynamic therapy for the treatment of esophageal carcinoma.^[68]^ Argon plasma coagulation therapy, which involves coagulating target tissues with high energy from argon ionization, is also an effective and successful method for precancerous lesions of the esophagus.^[69]^ There were no significant differences in the therapeutic effects of the 3 types of endoscopy. In clinical treatment, the choice of the 3 types of endoscopy depends mainly on the patient’s tolerance degree and operator’s proficiency. Studies have indicated that the 5-year survival rate of endoscopic treatment is > 90%.^[16]^

## 2. Limitations of endoscopy

Nevertheless, similar to all screening methodologies, the use of endoscopic diagnosis remains speculative without confirmatory randomized trial data. Early excision is not always beneficial because not all precancerous lesions progress, and treatment may cause severe complications and side effects. First, small artery hemorrhage and esophageal perforation are complications often encountered and need to be treated time.^[70,71]^ Second, the formation of stenosis is one of the implications after esophageal endoscopic mucosal dissection, which can be prevented by postoperative hormone application. Once the stenosis is formed, it can be treated by esophageal balloon dilation.^[72]^ In addition, Lugol chromoendoscopy is prohibited in patients with thyroid dysfunction and iodine allergy.^[73]^ Additionally, endoscopic treatment are forbidden in patients with obstruction, esophageal varices, ulcers, bleeding of the esophagus, serious heart disease, severe hypertension, laryngitis, and extreme physical weakness. In addition, it can cause discomfort, nausea, and vomiting. Therefore, several patients with no symptoms are reluctant to accept the detection, and some patients with local esophageal injury or general contraindications do not have the chance to receive this treatment.

In addition, populations with high ESCC incidence rates tend to be economically poor and medically underserved. Endoscopic screening and treatment of an entire population would be expensive and would require highly trained physicians and expensive equipment.^[74]^ Therefore, these diagnostic detection do not achieve the desired effect for economic or other reasons.

In conclusion, endoscopy dramatically improves the treatment of precancerous lesions and prevents the progression of precancerous lesions to ESCC, although with some limitations and complications.

### 2.1. Drug treatment development of esophageal precancerous lesions

Currently, there are no drugs available for the treatment of esophageal precancerous lesions. Laura et al investigated the chemoprevention of esophageal tumorigenesis using the dietary administration of lyophilized black raspberries.^[75]^ They found that lyophilized black raspberries inhibits N-nitrosomethylbenzylamine-induced esophageal tumorigenesis in F344 rats during the initiation and post-initiation phases of carcinogenesis. This study suggests that the development of drugs to reverse esophageal precancerous lesions is ongoing. On the other hand, the abnormal expression of biomarkers in esophageal lesions is also significant for the development of medical therapies. It is well known that specific antibodies against tumor-specific antigens can be employed as a target therapy in clinical treatment. However, drug treatment for esophageal precancerous lesions is still in its infancy, and its benefits need further exploration.

### 2.2. Living habits change for treating esophageal precancerous lesions

Esophageal squamous cell precancerous lesions can be transformed into cancer with risk factors, while once the risk factors are removed, precancerous lesions may also have opportunities to remain still or revert to normal tissues. Therefore, removal of this risk is also a good choice for treating precancerous lesions. Giving up smoking and alcohol, avoiding pickling and fungal infection, and maintaining a balanced diet, for example, are beneficial for patients with precancerous conditions.

## 3. Conclusions

Therapy for esophageal precancerous conditions plays a pivotal role in decreasing the incidence and mortality of ESCC. In conclusion, endoscopy greatly dramatically improves the treatment of precancerous lesions and prevents the progression of precancerous lesions to ESCC. Biomarkers provide opportunities for new diagnosis and treatment chances. Drugs and living habits may supply opportunities for treatment of esophageal precancerous lesions.

## Author contributions

**Conceptualization:** Xinran Chen.

**Data curation:** Tianjiao Wen.

**Formal analysis:** Xinran Chen.

**Funding acquisition:** Xinran Chen.

**Investigation:** Wei Wang.

**Methodology:** Xinran Chen.

**Resources:** Wei Wang.

**Software:** Wei Wang.

**Supervision:** Tianjiao Wen.

**Validation:** Wei Wang.

**Visualization:** Xinran Chen.

**Writing – original draft:** Tianjiao Wen.

**Writing – review & editing:** Tianjiao Wen.
